# Modulation of innate immune response to viruses including SARS-CoV-2 by progesterone

**DOI:** 10.1038/s41392-022-00981-5

**Published:** 2022-04-25

**Authors:** Shan Su, Duo Hua, Jin-Peng Li, Xia-Nan Zhang, Lei Bai, Li-Bo Cao, Yi Guo, Ming Zhang, Jia-Zhen Dong, Xiao-Wei Liang, Ke Lan, Ming-Ming Hu, Hong-Bing Shu

**Affiliations:** 1 Department of Infectious Diseases, Frontier Science Center for Immunology and Metabolism, Medical Research Institute, Zhongnan Hospital of Wuhan University, Wuhan University, Wuhan, 430071 China; 2 Department of Thyroid and Breast Surgery, Zhongnan Hospital of Wuhan University, Wuhan, 430071 China; 3 State Key Laboratory of Virology, College of Life Sciences, Wuhan University, Wuhan, 430071 China

**Keywords:** Innate immunity, Inflammation

## Abstract

Whether and how innate antiviral response is regulated by humoral metabolism remains enigmatic. We show that viral infection induces progesterone via the hypothalamic-pituitary-adrenal axis in mice. Progesterone induces downstream antiviral genes and promotes innate antiviral response in cells and mice, whereas knockout of the progesterone receptor PGR has opposite effects. Mechanistically, stimulation of PGR by progesterone activates the tyrosine kinase SRC, which phosphorylates the transcriptional factor IRF3 at Y107, leading to its activation and induction of antiviral genes. SARS-CoV-2-infected patients have increased progesterone levels, and which are co-related with decreased severity of COVID-19. Our findings reveal how progesterone modulates host innate antiviral response, and point to progesterone as a potential immunomodulatory reagent for infectious and inflammatory diseases.

## Introduction

Innate immunity is the first line of host defense against viral infection. Upon infection, viral components particularly their genomes or replication intermediates are sensed by distinct pattern-recognition receptors (PRRs) including Toll-like receptors (TLRs), RIG-I-like receptors (RLRs), and/or intracellular DNA sensors.^1^ Sensing of viral nucleic acids by PRRs triggers signaling cascades to induce transcription of type I interferons (IFNs), pro-inflammatory cytokines, and other downstream antiviral effector genes. The downstream cytokines and effectors inhibit viral replication or induce apoptosis of infected cells, leading to innate antiviral response.^2–8^

Different types of viruses are sensed by distinct PPRs. Upon infection of RNA virus, the invaded cytosolic viral RNA is sensed by the RNA helicase RIG-I or MDA5.^9,10^ Binding of RIG-1 or MDA5 to viral RNA triggers its recruitment to VISA (also called MAVS), a mitochondrion-associated adapter protein.^11,12^ VISA then recruits the adapter protein TRAF3, the TBK1 kinase, and the transcription factor IRF3. In this process, TBK1 phosphorylates IRF3 at multiple sites including S386 and S396, which causes IRF3 dimerization and translocation into the nucleus, leading to the ultimate induction of downstream cytokines and other antiviral effector genes.^13^

Upon infection of DNA virus, the invaded cytosolic viral DNA is sensed by the cyclic GMP-AMP (cGAMP) synthase (cGAS).^14,15^ cGAS can also recognize mis-located cellular genomic DNA or mitochondrial DNA released into the cytosol upon mitostress triggered by viral infection or other stress conditions.^16,17^ Upon sensing of DNA, cGAS utilizes GTP and ATP as substrates to synthesize cGAMP, which binds to the ER-located adapter protein MITA (also called STING).^18–20^ This triggers the translocation of MITA from the ER via Golgi apparatus to perinuclear punctate structures. In this process, MITA recruits TBK1 and IRF3, leading to IRF3 activation and induction of downstream antiviral genes.^20–22^

As a central transcriptional factor for induction of innate antiviral response, IRF3 needs to be fully activated in the early phase of viral infection, and then timely terminated to avoid excessive and harmful innate immune response.^23,24^ So far, various post-translational modifications, such as ubiquitination, sumoylation, and acetylation, have been reported to restrain IRF3 activity.^25–27^ In contrast, how IRF3 is optimally activated upon viral infection is not fully understood.

Viral infection not only triggers innate antiviral response, but also alters metabolic homeostasis in hosts.^28–30^ For example, viral infection changes the expression levels of rate-limiting enzymes involved in oxidative phosphorylation, sterol biosynthesis and redox homeostasis, leading to subsequent changes of various metabolic reactions in infected cells.^28–30^ Viral infection also causes changes of serum levels of neuroendocrine and other hormones, thereby affecting various physiological processes of the host.^31–33^ In recent years, several studies have revealed the mechanisms on how innate antiviral response is regulated by cellular metabolism such as cholesterol depletion,^34^ bile acid synthesis,^35^ lactate accumulation,^36^ and lipid oxidation.^37^ So far, how humoral metabolism regulates innate antiviral immunity is largely unknown.

Progesterone is an important humoral steroid hormone in mammals.^38^ In addition to its role in maintaining pregnancy of females, progesterone is also produced and functions in males. It has been reported that progesterone regulates neural development and T-cell differentiation regardless of genders.^39–42^ Progesterone is biosynthesized in specialized tissues, including adrenal gland and reproductive organs, and progesterone production is tightly regulated by the neuroendocrine hypothalamic-pituitary-adrenal (HPA) axis.^43,44^ Though progesterone is produced in specialized cells and tissues, it targets various types of cells in different tissues and organs to exert its diverse biological roles. Progesterone signals through PGR, a nuclear hormone receptor that directly induces transcription of downstream effector genes.^45^ In addition to its function as a nuclear hormone receptor, PGR can activate the proto-oncogene tyrosine kinase SRC rapidly in the cytoplasm of breast cancer and mammary epithelial cells upon progesterone stimulation.^46–48^

In this study, we identified progesterone as the mostly elevated humoral steroid hormone in virus-infected mice. Our results indicate that progesterone promotes innate antiviral response in vitro and in vivo. Mechanistically, the progesterone-PGR axis activates SRC, which then mediates phosphorylation of IRF3 at Y107. This phosphorylation of IRF3 primes for its association with TBK1 and self-association, leading to its activation and induction of downstream antiviral genes. Additionally, our studies also indicate that SARS-CoV-2-infected patients have increased serum progesterone levels, which are oppositely co-related with severity of the disease. Our findings reveal mechanisms on how progesterone metabolism modulates host innate antiviral response, and point to progesterone as a potential immunomodulatory reagent for infectious and inflammatory diseases.

## Results

### Viral infection increases serum progesterone level via the HPA axis in mice

**Fig. 1:**
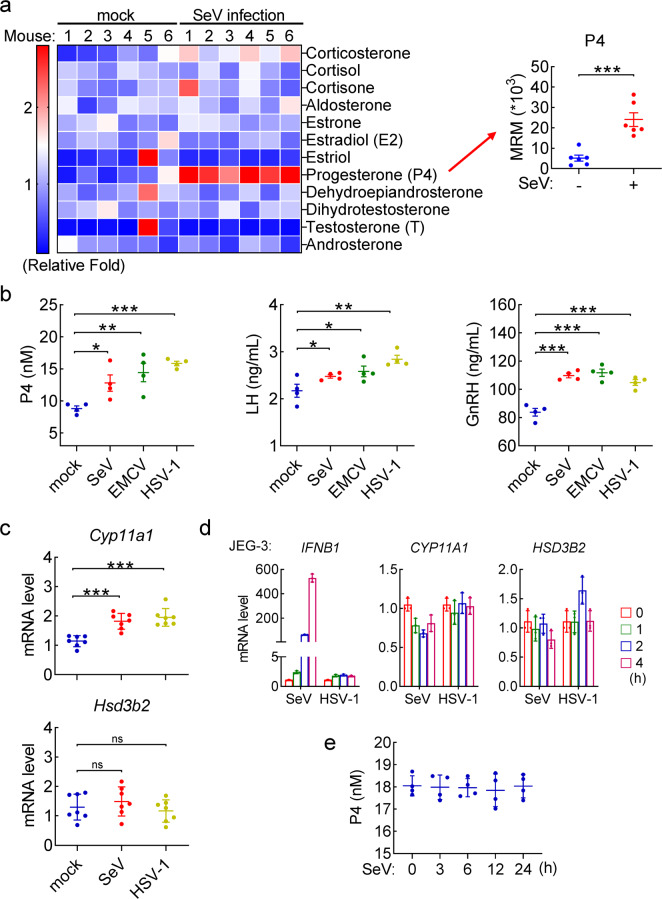
Viral infection increases progesterone level in cells and mice. **a** Targeted metabolomic analysis of steroid hormones in sera of SeV-infected mice. C57BL/6 male mice (*n* = 6 in each group) were left un-infected or infected intravenously with SeV for 3 h before the sera were collected and target metabolomic analysis was performed by liquid chromatography mass spectrometry (LC-MS). The data were then displayed by heatmap (the left panel), and the individual serum progesterone (P4) intensity in each sample was shown in the right histogram. MRM, multi reaction monitoring. **b** Measurement of neuroendocrine hormones in mice infected with different types of viruses. C57BL/6 male mice (*n* = 4 in each group) were left uninfected or infected intravenously with SeV for 3 h, EMCV for 2 h or HSV-1 for 6 h before sera were collected for measurements of progesterone (P4), GnRH and LH as indicated by ELISA assays. **c** Measurement of the mRNA levels of *Cyp11a1* and *Hsd3b2* genes. C57BL/6 male mice (*n* = 7 in each group) were left uninfected or infected intravenously with SeV for 3 h or HSV-1 for 6 h before their adrenal glands were collected for qPCR analysis of mRNA levels of *Cyp11a1* and *Hsd3b2* genes. **d** Measurement of the mRNA levels of *CYP11A1* and *HSD3B2* in human progesterone-producing cells. JEG-3 cells were left uninfected or infected with the indicated viruses for the indicated times before qPCR analysis of the mRNA levels of *CYP11A1* and *HSD3B2* genes. **e**. Effects of SeV infection on progesterone secretion in JEG-3 cells. JEG-3 cells were cultured in basic RPMI medium and then left uninfected or infected with SeV for the indicated times. The culture media were then collected for progesterone measurement by ELISA assays. *P < 0.05; **P < 0.01; ***P < 0.001; ns not significant

How innate antiviral immune response is regulated by humoral metabolites is largely unknown. Therefore, we performed a series of targeted metabolomic analysis of the sera from both control and virus-infected mice by liquid chromatography mass spectrometry. In these experiments, we used male mice due to their steadier humoral metabolic homeostasis which is affected by the menstrual cycle in female mice.^49^ These experiments led to our identification of progesterone (P4) as the mostly elevated steroid hormone in the sera of mice infected by Sendai virus (SeV), which is a RNA virus (Fig. 1a and Fig. S1a). We confirmed the increase of P4 in the sera of mice infected with SeV as well as the RNA virus encephalomyocarditis virus (EMCV) and the DNA virus herpes simplex virus-1 (HSV-1) (Fig. 1b), suggesting that humoral progesterone levels in mice are increased following infection of different types of viruses.

In mammals, secretion of P4 is regulated by the neuroendocrine HPA axis.^43,44^ The hypothalamus-secreted gonadotropin-releasing hormone (GnRH) stimulates the pituitary to produce luteinizing hormone (LH), which then targets the adrenal and reproductive organs to promote transcription of *CYP11A1*, the rate-limiting metabolic enzyme for P4 biosynthesis. We examined the levels of GnRH and LH in sera of both control and virus-infected mice, and found that viral infection increased the levels of both GnRH and LH (Fig. 1b). Additionally, the mRNA level of *Cyp11a1* but not *Hsd3b2* (a non-rate-limiting metabolic enzyme for P4 biosynthesis) gene was increased in the adrenal gland of virus-infected in comparison to control mice (Fig. 1c and S1b). In the same experiments, the mRNA levels of enzyme genes involved in biosynthesis of other sex steroid hormones was also examined^50,51^ (Fig. S1b). The results showed that transcription of *Cyp17a1* (the rate-limiting metabolic enzyme for testosterone and estradiol biosynthesis) gene was induced upon viral infection (Fig. S1c), but the mRNA level of *Cyp19a1* (another rate-limiting metabolic enzyme for estradiol biosynthesis) gene was hardly affected by virus (Fig. S1c). Interestingly, the mRNA level of *Hsd17b1* gene (a non-rate-limiting metabolic enzyme for both testosterone and estradiol biosynthesis) was markedly down-regulated following viral infection (Fig. S1c), which might account for the unchanged serum levels of testosterone and estradiol in SeV-infected mice (Figs. 1a and S1a). Furthermore, we found that viral infection had no marked effects on transcription of *CYP11A1* and *HSD3B2* genes (Fig. 1d) as well as P4 production (Fig. 1e) in cultured chorionic epithelial cell line JEG-3, which naturally produces P4.^52^ In these experiments, viral infection induced transcription of *IFNB1* gene in these cells (Fig. 1d), suggesting that the cells are successfully infected. Taken together, these results suggest that viral infection does not directly induces P4 production but increases its humoral levels via the HPA axis in mice.

### The progesterone-PGR axis modulates innate antiviral response in vivo

**Fig. 2:**
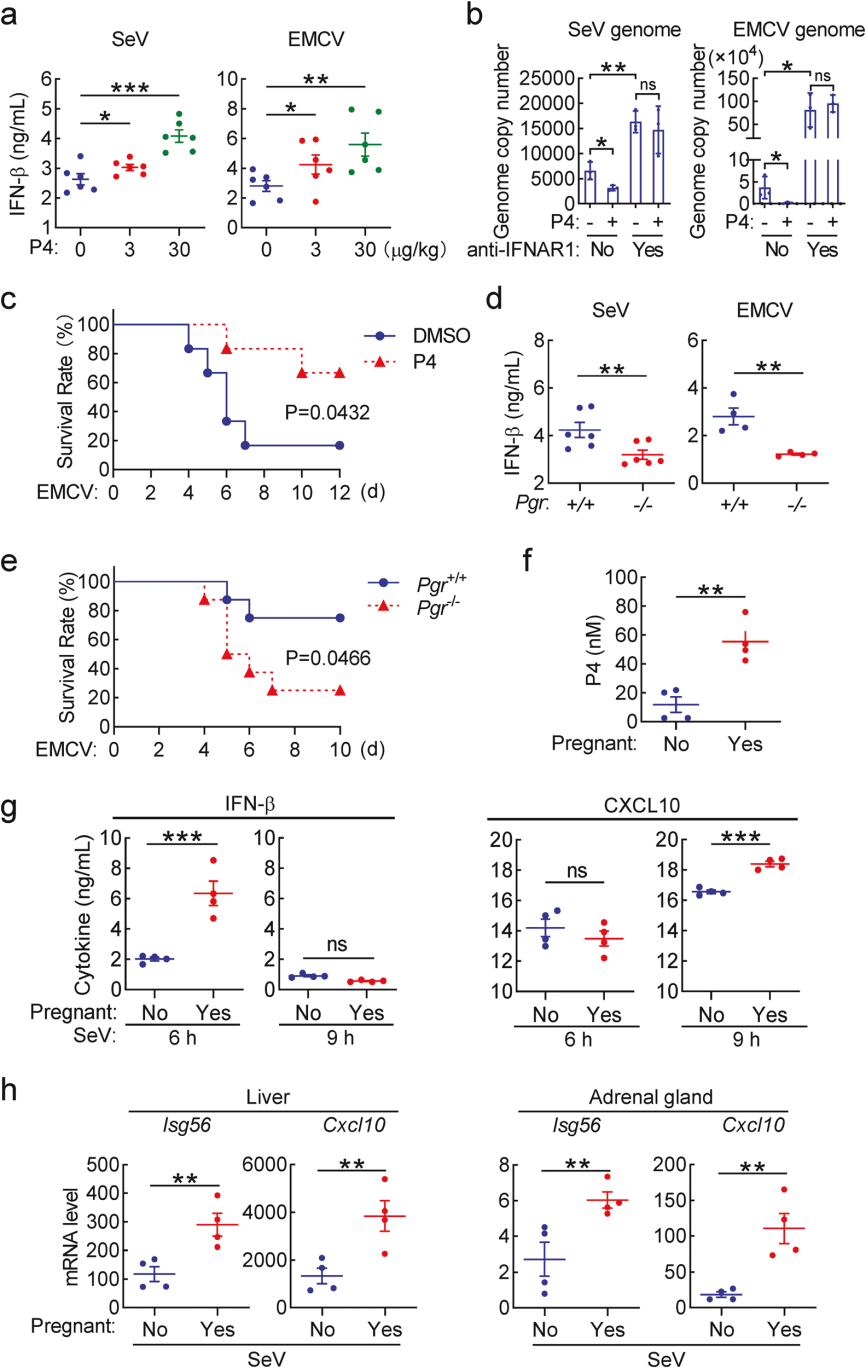
The progesterone-PGR axis is required for efficient innate antiviral response in vivo. **a** Effects of progesterone on virus-induced IFN-β production in the sera of mice. Mice (male, *n* = 6 in each group) were injected intravenously with the indicated doses of P4 for half an hour, and then infected with SeV or EMCV (1 × 10^8^ pfu) for 6 h before measurement of serum IFN-β by ELISA assays. **b** Effects of progesterone on viral replication in mice. Mice (8-week-old male, *n* = 3) were intraperitoneally (i.p.) treated with a neutralizing antibody (nAb) against IFNAR-1 (500 μg/mouse) or PBS, and then injected intravenously with DMSO or P4 (30 μg/kg) for half an hour, followed by intraperitoneal injection of SeV or EMCV (1 × 10^8^ pfu) and repeated antibody treatments as described in the Materials and Methods section. Viral genomic copy numbers in the spleens of SeV-infected mice or the brains of EMCV-infected mice were quantified by qPCR. **c** Effects of progesterone on virus-induced death of mice. Mice (8-week-old male, *n* = 6) were injected intravenously with DMSO or P4 (30 μg/kg) for half an hour, and then infected intraperitoneally with EMCV (1 × 10^7^ pfu). The survival rates were monitored for twelve days. **d** Effects of Pgr-deficiency on virus-induced production of serum cytokines. *Pgr*^*+/+*^ and *Pgr*^−*/*−^ male mice were infected intravenously with SeV (*n* = 6 mice in each group) or EMCV (1 × 10^8^ pfu, *n* = 4 mice in each group) for 6 h before serum cytokines were measured by ELISA. **e** Effects of Pgr-deficiency on EMCV-induced death of mice. *Pgr*^*+/+*^ and *Pgr*^*−/*−^ mice (*n* = 8 per strain, 8-week-old male) were infected intraperitoneally with EMCV (1 × 10^7^ pfu per mice), and the survival rates of mice were monitored for ten days. **f** Progesterone levels in pregnant mice. Sera from control and two-week pregnant female mice (*n* = 4 in each group) were collected for progesterone measurement by ELISA assays. **g** Levels of serum IFN-β and CXCL10 in virus-infected pregnant mice. The control and two-week pregnant female mice (*n* = 4 in each group) were infected intravenously with SeV for the indicated times before serum IFN-β and CXCL10 were measured by ELISA assays. **h** The mRNA levels of antiviral genes in different tissues of virus-infected pregnant mice. The control and two-week pregnant female mice (*n* = 4 in each group) were infected intravenously with SeV for 6 h, and then the indicated tissues were collected for qPCR analysis of mRNA levels of the indicated genes. *P < 0.05; **P < 0.01; ***P < 0.001; ns not significant

Since viral infection causes increase of humoral progesterone, we investigated whether progesterone regulates innate antiviral response in vivo. We found that administration of exogenous P4 by tail vein injection potentiated both SeV- and EMCV-induced serum production of IFN-β, a central cytokine in innate antiviral immune response (Figs. 2a and S2a). In the same experiments, serum TNFα and IL-6 in virus-infected mice were not markedly changed by P4 treatment (Fig. S2b–d), suggesting that P4 has a selective role in promoting IFN-β production. Exogenous P4 administration dramatically suppressed SeV and EMCV replication in mice (Fig. 2b). Injection of mice with an antagonistic antibody for type I IFN receptor IFNAR-1 potentiated viral replication and rescued P4-induced inhibition of viral replication (Fig. 2b). These results suggest that P4 inhibits viral replication in mice in a type I IFN-dependent manner. In addition, exogenous P4 protected mice from EMCV-induced death (Fig. 2c). These results suggest that progesterone promotes innate antiviral response in mice.

Progesterone exerts most of its biological functions via the progesterone receptor (PGR) in mammals.^53^ To investigate whether endogenous progesterone-PGR axis plays a role in innate antiviral response in vivo, we generated *Pgr*-knockout mice (Fig. S3a) and confirmed by both genotyping (Fig. S3b) and qPCR analysis of *Pgr* mRNA in mouse primary cells including bone marrow-derived dendric cells (BMDCs) and bone marrow-derived macrophages (BMDMs) (Fig. S3c). These experiments also indicated that *Pgr* mRNA was detected in BMDCs but not BMDMs (Fig. S3c). Furthermore, we observed decrease of serum P4 level in *Pgr*-knockout mice regardless of the genders (Fig. S3d), suggesting that PGR is involved in transcriptional regulation of genes related to P4 biosynthesis.^54,55^ Next, we found that Pgr-deficiency markedly inhibited serum levels of IFN-β induced by either SeV or EMCV in male mice (Fig. 2d), though the basal serum levels of IFN-β were comparable between wide-type and *Pgr*-knockout male and female mice (Fig. S3e). Pgr-deficiency also caused an earlier on-set of death and a lower survival rate of mice infected with EMCV (Fig. 2e). These results suggest that the progesterone-PGR axis plays an important role in efficient host defense against viral infection in vivo.

High humoral progesterone is a biomarker for pregnancy of females.^56^ We examined the potential difference in innate antiviral immunity between pregnant and non-pregnant mice after viral infection. As expected, high serum progesterone levels were identified in pregnant (Yes) but not non-pregnant (No) mice (Fig. 2f). In addition, the levels of serum IFN-β and CXCL10 induced by SeV infection in pregnant mice were markedly higher than in non-pregnant mice (Fig. 2g). The mRNA levels of downstream antiviral genes *Isg56* and *Cxcl10* in the livers and adrenal glands of pregnant mice were markedly higher than that of non-pregnant mice after SeV infection (Fig. 2h). These results suggest that pregnant mice have heightened innate antiviral response.

### The progesterone-PGR axis modulates innate antiviral response via SRC activation

**Fig. 3:**
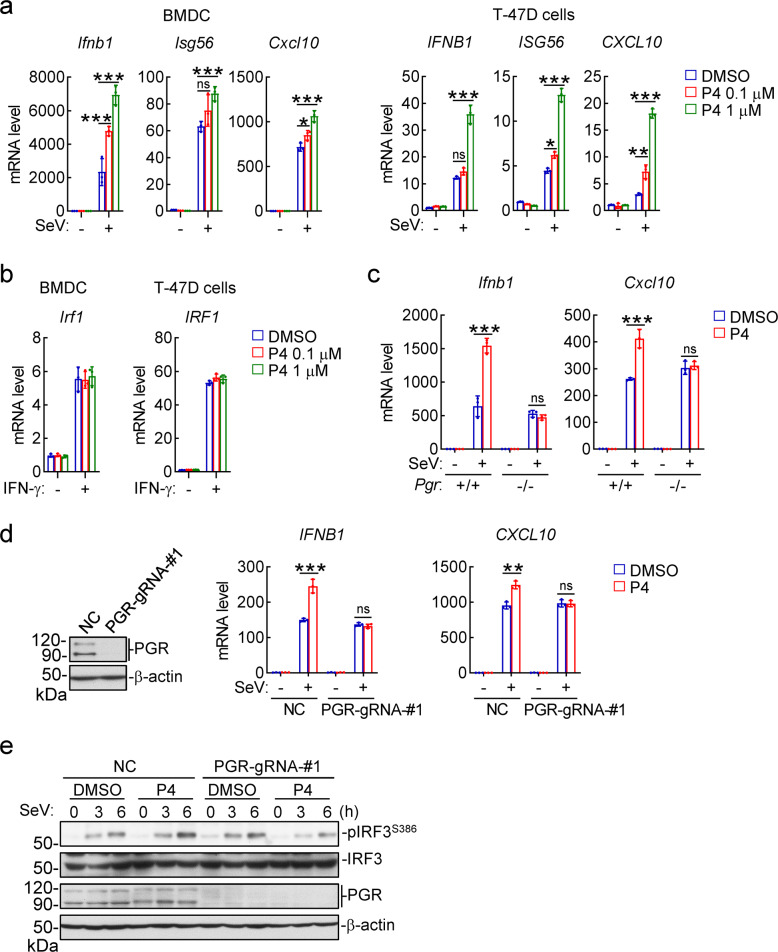
The progesterone-PGR axis promotes innate antiviral signaling. **a** Effects of progesterone on transcription of antiviral genes. BMDCs or T-47D cells were treated with the indicated doses of P4 for 1 h and then infected with SeV for 6 h before qPCR analysis of mRNA levels of the indicated antiviral genes. **b** Effects of progesterone on IFN-γ-induced transcription of *IRF1* gene. BMDCs or T-47D cells were treated with the indicated doses of P4 for 1 h and then stimulated with IFN-γ (100 ng/ml) for 6 h before qPCR analysis of *IRF1* mRNA level. **c** Effects of progesterone on transcription of antiviral genes in *Pgr*^*+/+*^ and *Pgr*^−*/*−^ BMDCs. *Pgr*^*+/+*^ and *Pgr*^−*/−*^ BMDCs were treated with P4 (1 μM) for 1 h and then infected with SeV for 6 h before qPCR analysis of mRNA levels of the indicated genes. **d** Effects of progesterone on transcription of antiviral genes in PGR-knockout cells. The control and PGR-knockout T-47D cells were treated with P4 (1 μM) for 1 h and then infected with SeV for 6 h before qPCR analysis of mRNA levels of the indicated genes. **e** Effects of progesterone treatment on virus-induced activation of IRF3 in control and PGR-knockout T-47D cells. The control and PGR-knockout T-47D cells were treated with P4 (1 μM) for 1 h, and then left uninfected or infected with SeV for the indicated times before immunoblotting analysis with the indicated antibodies. Data shown in a-d are mean ± SD (*n* = 3) from one representative experiment, which was repeated for at least two times with similar results. *P < 0.05; **P < 0.01; ***P < 0.001

We next investigated how progesterone promotes innate antiviral response. We found that P4 treatment dose-dependently potentiated SeV-induced transcription of antiviral *IFNB1*, *ISG56* and *CXCL10* genes in mouse primary bone marrow-derived dendric cells (BMDCs) and human breast cancer T-47D cells (Fig. 3a), both expresses PGR (Fig. S3c).^57^ In similar experiments, P4 had no marked effects on IFN-γ-induced transcription of *IRF1* gene in these cells (Fig. 3b). Knockout of PGR impaired the ability of P4 to potentiate SeV-induced transcription of *IFNB1* and *CXCL10* genes in both BMDCs (Fig. 3c) and T-47D cells (Fig. 3d). In addition, P4 treatment potentiated SeV-induced phosphorylation of IRF3 at Ser386 (p-IRF3^S386^, a hallmark for its activation) in control but not PGR-knockout T-47D cells (Fig. 3e). These results suggest that the progesterone-PGR axis promotes innate antiviral signaling in both human and mouse cells.

**Fig. 4:**
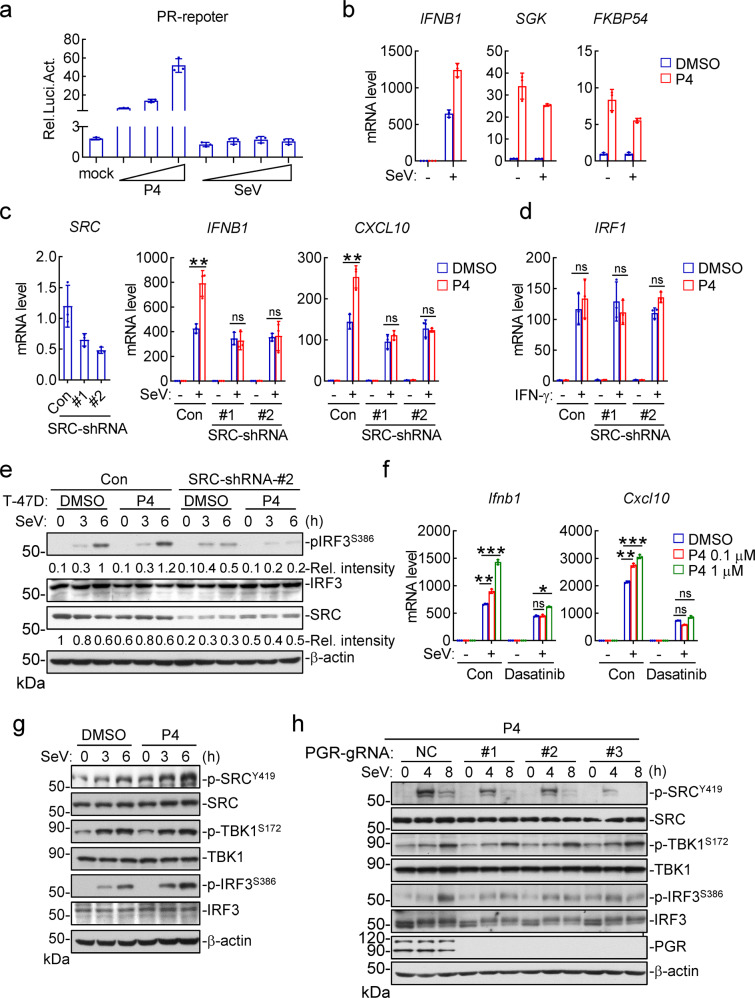
The progesterone-PGR axis promotes innate antiviral response via activation of SRC. **a** Effects of progesterone or viral infection on PR activation. The PGR-overexpressed HEK293 cells were transfected with PR reporter for 24 h, and then left untreated or treated with increased doses of progesterone (0.01, 0.1, 1 μM) or infected with different doses of SeV for 10 h before luciferase assays. **b** Effects of progesterone and viral infection on transcription of nuclear PGR-targeted genes. T-47D cells were treated with DMSO or P4 (1 μM) for 1 h and then left uninfected or infected with SeV for 6 h before qPCR analysis of mRNA levels of the indicated genes. **c** Effects of progesterone on virus-induced transcription of antiviral genes in SRC-knockdown cells. The control and SRC-knockdown T-47D cells were treated with P4 (1 μM) for 1 h and then left uninfected or infected with SeV for 6 h before qPCR analysis of mRNA levels of the indicated genes. The knockdown efficiency of SRC was shown by qPCR analysis of mRNA level in the left panels. **d** Effects of progesterone on IFN-γ-induced transcription of *IRF1* genes in SRC-knockdown cells. The control and SRC-knockdown T-47D cells were treated with P4 (1 μM) for 1 h and then left untreated or treated with IFN-γ (100 ng/ml) for 6 h before qPCR analysis of *IRF1* mRNA level. **e** Effects of progesterone on virus-induced phosphorylation events in SRC-knockdown T-47D cells. The control and SRC-knockdown T-47D cells were treated with P4 (1 μM) for 1 h, and then left uninfected or infected with SeV for the indicated times before immunoblotting analysis with the indicated antibodies. The relative intensities of p-IRF3^S386^ and SRC (normalized to β-actin) were analyzed by ImageJ. **f** Effects of SRC inhibitor on progesterone-potentiated transcription of antiviral genes. BMDCs were pretreated with DMSO or Dasatinib (SRC inhibitor, 10 μM) and together with the indicated doses of P4 for 1 h. The cells were then left uninfected or infected with SeV for 6 h before qPCR analysis of the mRNA levels of *Ifnb1* and *Cxcl10* genes. **g** Effects of progesterone on virus-induced activation of SRC, TBK1 and IRF3. T-47D cells were treated with P4 (1 μM) for 1 h, and then left un-infected or infected with SeV for the indicated times before immunoblotting analysis with the indicated antibodies. **h** Effects of PGR-deficiency on SeV-induced activation of SRC, TBK1, and IRF3. The control and PGR-KO T-47D cells were cultured in P4-containing medium, and then left uninfected or infected with SeV for the indicated times before immunoblotting analysis with the indicated antibodies. Data shown in a-d, f are mean ± SD (*n* = 3) from one representative experiment, which was repeated for at least two times with similar results. *P < 0.05; **P < 0.01; ***P < 0.001; ns, not significant

It has been well established that PGR acts either as a nuclear steroid receptor for transcriptional induction or as a cytoplasmic activator of the tyrosine kinase SRC.^45,46^ In reporter assays, P4 activated transcription driven by the PGR-responsive element (PR) in a dose-dependent manner (Fig. 4a). In similar experiments, SeV infection had no marked effects on PR activity (Fig. 4a). Consistently, P4 treatment potentiated SeV-induced transcription of *IFNB1* gene, whereas SeV infection failed to potentiate P4-induced transcription of nuclear PGR-targeted genes including *SGK* and *FKBP54*^54,58,59^ (Fig. 4b). These results suggest that viral infection has no marked effects on the transcriptional activity of PGR.

We next examined whether PGR regulates innate antiviral response via its downstream tyrosine kinase SRC.^46^ We found that P4 potentiated SeV-induced transcription of *IFNB1* and *CXCL10* genes in control but not SRC-knockdown T-47D cells (Fig. 4c). In similar experiments, P4 had no marked effects on IFN-γ-induced transcription of *IRF1* in these cells (Fig. 4d). Consistently, P4 potentiated SeV-induced phosphorylation of IRF3^S386^ in control but not SRC-knockdown T-47D cells (Fig. 4e). Inhibition of SRC kinase activity with a selective inhibitor Dasatinib^60^ impaired the potentiation effects of P4 on SeV-induced transcription of the antiviral genes *Ifnb1* and *Cxcl10* in BMDCs (Fig. 4f). In addition, we found that SeV infection induced phosphorylation of SRC at Y419 (p-SRC^Y419^, a hallmark for its activation) to a limited degree, which was potentiated by P4 treatment in T-47D cells (Fig. 4g). In these experiments, SeV-induced phosphorylation of IRF3^S386^ was also potentiated by P4 (Fig. 4g). Furthermore, knockout of PGR inhibited phosphorylation of SRC^Y419^ and IRF3^S386^ induced by SeV infection and P4 stimulation in T-47D cells (Fig. 4h). Coimmunoprecipitation experiments indicated that endogenous PGR was weakly associated with SRC in P4- but not DMSO-treated T-47D cells, which was further enhanced after SeV infection (Fig. S4). Taken together, these results suggest that P4 induces PGR-dependent activation of SRC, which promotes virus-triggered activation of IRF3 as well as induction of downstream antiviral genes.

### SRC mediates tyrosine phosphorylation of IRF3 to promote its activation

**Fig. 5:**
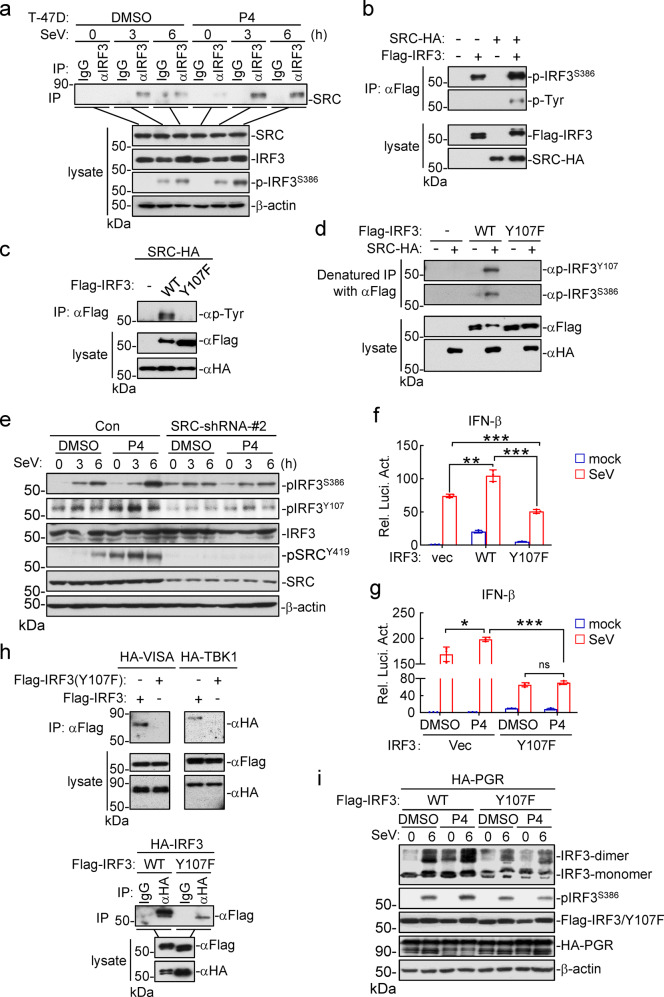
SRC mediates phosphorylation of IRF3 at Y107 and its activation. **a** Effects of progesterone on endogenous association of IRF3 with SRC. T-47D cells were treated with P4 (1 μM) for 1 h, and then left uninfected or infected with SeV for the indicated times before coimmunoprecipitation and immunoblotting analysis with the indicated antibodies. **b** Effects of SRC on tyrosine phosphorylation of IRF3. HEK293 cells were transfected with the indicated plasmids for 24 h before coimmunoprecipitation and immunoblotting analysis with the indicated antibodies. **c** SRC does not mediate IRF3^Y107F^ phosphorylation. HEK293 cells were transfected with the indicated plasmids for 24 h before coimmunoprecipitation and immunoblotting analysis with the indicated antibodies. **d** SRC mediates phosphorylation of IRF3^Y107^. HEK293 cells were transfected with the indicated plasmids for 24 h. The cell lysates were denatured by 1% SDS, which were then diluted with regular lysis buffer till the final concentration of SDS reached to 0.1%. The lysates were then immunoprecipitated with a Flag antibody, and then the immunoprecipitation were analyzed by immunoblots with the indicated antibodies. **e** Effects of progesterone on virus-induced phosphorylation of IRF3^Y107^ in SRC-knockdown cells. The control and SRC-knockdown T-47D cells were treated with P4 (1 μM) for 1 h, and then left uninfected or infected with SeV for the indicated times before immunoblotting analysis with the indicated antibodies. **f** Effects of IRF3 and IRF3^Y107F^ on SeV-induced activation of the IFN-β promoter. HEK293 cells were transfected with the indicated plasmids for 24 h, and then infected with SeV for 12 h before luciferase assays. Data shown are mean ± SD (*n* = 3). *P < 0.05; **P < 0.01; ***P < 0.001. **g** Effects of IRF3^Y107^ mutant on progesterone-potentiated activation of the IFN-β promoter. The PGR-overexpressed HEK293 cells were transfected with the indicated plasmids for 24 h, and then left treated or untreated with P4 (1 μM) for 1 h followed by SeV infection for 12 h before luciferase assays. Data shown are mean ± SD (*n* = 3). *P < 0.05; **P < 0.01; ***P < 0.001. **h**. Effects of IRF3 Y107 mutation on its association with VISA and TBK1 or self-association. HEK293 were transfected with the indicated plasmids for 24 h before coimmunoprecipitation and immunoblotting analysis with the indicated antibodies. **i** Effects of IRF3 Y107 mutation on its dimerization. HEK293 were transfected with the indicated plasmids for 24 h, and then left treated or untreated with P4 (1 μM) for 1 h followed by SeV infection for 6 h. The cells were lysed for native PAGE and immunoblotting analysis with the indicated antibodies

In the above experiments, we noticed that SeV-induced phosphorylation of IRF3^S386^ but not phosphorylation of TBK1^S172^ (p-TBK1^S172^, a hallmark for TBK1 activation) was increased by P4 treatment (Fig. 4g) or decreased by knockout of PGR (Fig. 4h). Since TBK1 acts at upstream of IRF3 in innate antiviral signaling, these results suggest that the progesterone-PGR-SRC axis regulates innate antiviral signaling at IRF3 level. Endogenous coimmunoprecipitation experiments indicated that viral infection induced association of IRF3 with SRC, and P4 treatment further enhanced their association (Fig. 5a). In addition, overexpression of SRC catalyzed tyrosine phosphorylation of IRF3 (Fig. 5b). We next analyzed potential phosphorylation sites of IRF3 in both SRC-overexpressing and control cells by mass spectrometry. The results indicated that Y107 and Y292 of human IRF3 were two candidate residues targeted by SRC (Fig. S5a), and sequence alignments indicated that Y107 but not Y292 is conserved from murine to human (Fig. S5b). Mutation of IRF3 Y107 to phenylalanine (F) impaired SRC-mediated tyrosine phosphorylation of IRF3 in mammalian overexpression system (Fig. 5c). We made an antibody specific for Y107-phosphorylated IRF3 (Fig. S5c). With this antibody, we detected a signal of p-IRF3^Y107^ when wild-type human IRF3 but not IRF3^Y107F^ was co-transfected with SRC (Fig. 5d). In these experiments, SRC also caused phosphorylation of S386 in wild-type IRF3 but not IRF3^Y107F^ (Fig. 5d), indicating that SRC-mediated tyrosine phosphorylation of IRF3^Y107^ is required for its activation. Furthermore, P4 increased phosphorylation of endogenous IRF3^Y107^ and SeV-induced phosphorylation of IRF3^S386^, which was not seen in SRC-knockdown T-47D cells (Fig. 5e). Functionally, overexpression of wild-type IRF3 potentiated SeV-induced activation of the IFN-β promoter, whereas overexpression of IRF3^Y107F^ inhibited it (Fig. 5f). P4 treatment also potentiated SeV-induced activation of the IFN-β promoter, whereas overexpression of IRF3^Y107F^ inhibited it (Fig. 5g). Collectively, these results suggest that the P4-PGR-SRC axis mediates tyrosine phosphorylation of IRF3^Y107^ to promote its activation upon viral infection.

We further investigated how phosphorylation of IRF3^Y107^ regulates its activity. Structural studies have shown that IRF3 is consisted of the N-terminal DNA-binding domain (DBD, aa1-104), a middle flexible linker (MFL, aa105-198), an IRF3-associated domain (IAD, aa199-375) and the C-terminal repressor domain (RD, aa376-427).^61^ It has also been demonstrated that IRF3 is activated via three steps: unlock of its intramolecular autoinhibition to associate with other signal molecules (eg, VISA and TBK1), its dimerization and translocation into nucleus for transcriptional induction of antiviral genes.^62^ Since Y107 is located at the adjacent site of DBD and MFL of IRF3, we hypothesized that phosphorylation of IRF3^Y107^ may cause its conformational changes which affect its activation. Coimmunoprecipitation experiments showed that IRF3^Y107F^ lost its ability to associate with VISA and TBK1 (Fig. 5h). IRF3^Y107F^ also had dramatically reduced ability to self-associate in comparison to wild-type IRF3 (Fig. 5h). Consistently, IRF3^Y107F^ had reduced dimerization after SeV infection either with or without P4 treatment (Fig. 5i). These results suggest that phosphorylation of IRF3^Y107^ unlocks its intramolecular autoinhibition and promotes its activation. Additionally, we found that SRC interacted with the C-terminus (aa141-427) of IRF3 by domain mapping experiments (Fig. S6a). Based on our study and the previous reports on the VISA-TBK1-IRF3 complex,^63,64^ we propose a model on how SRC-mediated tyrosine phosphorylation of IRF3 at Y107 unlocks its intermolecular autoinhibition and primes its activation (Fig. S6b).

### Progesterone levels in SARS-CoV-2-infected patients

**Fig. 6:**
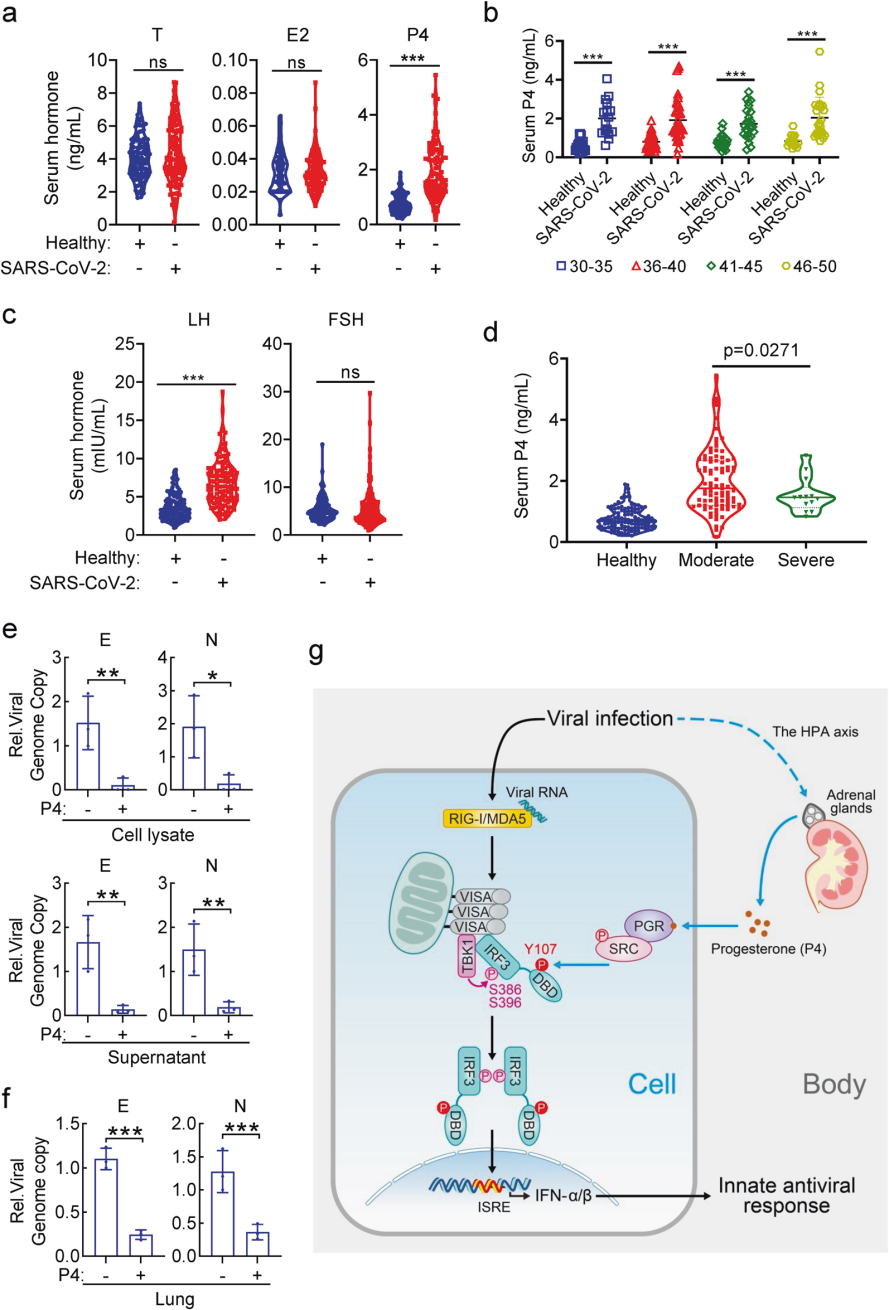
SARS-CoV-2 infection increases serum progesterone levels in COVID-19 patients. **a**–**c** Analysis of sex-related hormones in healthy individuals and SARS-CoV-2-infected patients. The measurements of testosterone (T), estradiol (E2) and progesterone (P4) are shown in (**a**). The age-classified data of P4 are shown in (**b**). The measurements of LH and FSH levels are shown in (**c**). *P < 0.05; **P < 0.01; ***P < 0.001; ns, not significant. **d** Correlation analysis between progesterone levels and severity of symptoms of COVID-19. **e** Effects of progesterone on replication of SARS-CoV-2 in Calu-3 cells. Calu-3 cells were treated with P4 (1 μM) for 1 h and then infected with SARS-CoV-2 (MOI = 0.01) for 48 h. Total RNAs in both cell lysate and the supernatant was collected to detect the envelope (**e**) and nucleocapsid (N) RNA of SARS-CoV-2 via TaqMan-qRT-PCR. **f** Effects of progesterone on replication of SARS-CoV-2 in mice. The K18-hACE2 transgenic mice (*n* = 3 in each group, 8-week-old male) were injected intraperitoneally (i.p.) with DMSO or P4 (30 μg/kg) for half an hour, and then intranasally infected with 250 PFU of SARS-CoV-2 in a total volume of 50 μl DMEM. Mice were then continued to be administered with DMSO or P4 daily until five days post-challenge. Viral genomic copy numbers in the lungs of infected mice were quantified by qPCR. **g** A work model for modulation of innate antiviral response by humoral progesterone

Recently, it has been reported that pregnant women have increased rates of progressing into severe COVID-19 symptoms after SARS-CoV-2 infection.^65^ We collected and analyzed sex hormone-testing reports of SARS-CoV-2-infected male patients (*n* = 104) and the healthy males (*n* = 107). As shown in Fig. 6a, progesterone levels (1.93 ± 0.99 ng/mL) in SARS-CoV-2-infected patients were ~3-fold higher to the healthy individuals (0.76 ± 0.34 ng/mL), whereas the other tested sex hormones including testosterone (T) and estradiol (E2) were comparable between the two groups (Fig. 6a). SARS-CoV-2 infection increased serum P4 levels in all age groups (Fig. 6b). In addition, patients infected by SARS-CoV-2 had increased serum LH levels (Fig. 6c), which was similar to our observation in virus-infected mice (Fig. 1c). Furthermore, statistical results showed that patients with severe symptoms (*n* = 13) displayed significant lower levels of serum P4 than those with moderate symptoms (*n* = 89) (Fig. 6d), suggesting a negative correlation between serum P4 level and the severity of COVID-19 symptoms. These results are consistent with an important role of progesterone in host innate antiviral response.

To confirm the potential role of P4 in modulating SARS-CoV-2 infection, we examined the effects of P4 treatment on SARS-CoV-2 replication by detecting the copy numbers of the viral genome with two specific probes for either envelope (E) or nucleocapsid (N) RNA of SARS-CoV-2.^66^ The results showed that P4 treatment markedly down-regulated the copy numbers of SARS-CoV-2 in cultured Calu-3 lung epithelial cells (Fig. 6e). Administration of P4 also down-regulated SARS-CoV-2 replication in the lung of K18-hACE2 transgenic mice (Fig. 6f). These results suggest that P4 has an inhibitory role on SARS-CoV-2 replication.

## Discussion

Several studies have revealed that viral infection causes changes of intracellular metabolic pathways, which in turn regulate innate antiviral response.^34–37^ However, how humoral metabolism regulates host innate antiviral response remains enigmatic. In this study, we found that viral infection induces humoral progesterone, which plays a critical role in promoting efficient innate antiviral response.

To investigate whether and how humoral hormones regulate innate antiviral response, we performed targeted metabolomics analysis of the sera of infected mice. These efforts identified progesterone as the mostly increased among tested humoral hormones after viral infection in mice. Viral infection also caused increases of GnRH and LH, which acts upstream to regulate the level of progesterone in vivo.^67,68^ Analysis of the sex-related hormone-testing reports of SARS-CoV-2-infected patients and healthy individuals indicated that SARS-CoV-2 infection also increased serum progesterone and LH levels. These results suggest that increase of humoral progesterone level via the HPA axis following viral infection is a conserved biological function from mouse to human. Future studies are needed to determine how viral infection activates the HPA axis. It is possible that the HPA axis is directly activated by viral infection of certain peripheral or central neurons in hypothalamus, or indirectly activated by virus-induced effects.

By studying with both mouse and human cells, as well as gene-knockout mice, we demonstrate that humoral progesterone facilitates innate antiviral response, and progesterone inhibits viral replication in a type I IFN-dependent manner and protects mice from virus-induced death. Several lines of evidences suggest that progesterone promotes innate antiviral response via PGR-mediated activation of SRC, which subsequently phosphorylates and activates IRF3 as illustrated in Fig. 6g. Firstly, either PGR- or SRC-deficiency impaired progesterone-triggered potentiation of IRF3 activation and antiviral gene expression following viral infection. Second, SRC interacted with and mediated phosphorylation of IRF3^Y107^. Third, phosphorylation of IRF3^Y107^ by SRC was important for IRF3 association with TBK1 and its self-association, as well as induction of IFN-β upon viral infection. Sequence analysis suggests that IRF3 Y107 is conserved from mouse to human, and located at the junction of the N-terminal DNA-binding domain and the middle flexible linker of IRF3. It is possible that phosphorylation of IRF3^Y107^ causes its conformational changes which unlock its autoinhibition and promote its phosphorylation at S386/S396 and activation by TBK1. Our current study suggests for the first time that phosphorylation of IRF3^Y107^ is essential for priming its activation. We noticed that IRF3^Y107^ is basally phosphorylated in un-infected cells and which is increased following viral infection and progesterone stimulation. It is possible that other stimuli that can activate SRC are also able to modulate innate antiviral response.

In our study, we found that except for the RNA viruses SeV and EMCV, the DNA virus HSV-1 also induced progesterone. In addition, progesterone also potentiated HSV-1-induced transcription of downstream antiviral genes. This is consistent with the notion that IRF3 is also a critical mediator of innate immune response to DNA virus, and suggests that progesterone modulates innate immune response to both RNA and DNA viruses. Our findings reveal a conserved and common regulatory mechanism of innate antiviral response by humoral progesterone. Systemic lupus erythematosus (SLE) is characterized by multi-system immune-mediated injury in the setting of autoimmunity to nuclear antigens with strong female bias.^69,70^ Since enhanced type I IFN signal is associated with SLE, it would be interesting to explore whether progesterone-mediated potentiation of type I IFN production is related to the higher risk of SLE in females.^71^

Sex differences in COVID-19 vulnerability and clinical manifestations have been observed by various studies. It has been well established that SARS-CoV-2-infected males have significantly higher rates of progressing into severe diseases, and male-to-female fatality ratios obtained from different countries are between 1.7-1.8.^72,73^ Notably, the male fatality predominance is mostly pronounced in the middle age group and linearly declined in the higher age groups,^72^ suggesting that the potential female’s protective factors for COVID-19 decline during aging. Our discovery that progesterone is induced following viral infection and which promotes innate antiviral response provides an explanation on the observations that SARS-CoV-2-infected females have reduced severity and fatality in comparison to males. Our study indicates that the progesterone levels are negatively co-related with the severity of COVID-19. It is possible that heightened innate immune response in SARS-CoV-2-infected patients with higher levels of progesterone contributes to the alleviated symptoms of COVID-19. It has been reported that pregnant women, who have a very high level of progesterone, have an increased requirement of intensive care unit admission after infection of SARS-CoV-2.^65^ Previously, it has been demonstrated that high level of progesterone has anti-inflammatory effects by inhibiting NF-κB pathways and T-cell-mediated adaptive immune responses.^42,74^ Since viral diseases are caused by both viral factors and improperly regulated inflammatory/immune responses, and progesterone has pleiotropic roles in modulating physiological functions and immunological responses, the eventual beneficial or harmful effects of progesterone on viral-infected patients may be dictated by its levels, host status and timing of viral infection. Nevertheless, our study suggests that progesterone may serve as a potential immunomodulatory agent for infectious diseases such as COVID-19 by both promoting innate antiviral response and restraining excessive inflammatory response.

## Materials and methods

### Reagents, antibodies, viruses, and cells

Progesterone (HY-N0437) and Dasatinib (HY-10181) (MedChemExpress); IFN-γ (300-02, Peptech); Anti-mouse IFNAR-1-in vivo (clone MAR1-5A3; Selleck A2121); ELISA kits for progesterone (582601, Cayman Chemical), GnRH (MM-0506M1) and LH (MM-44039M1) (MEIMAIN), murine IFN-β(42400, PBL), murine CXCL10 (EMC121, Neobioscience), murine TNFα (430904, BioLegend), murine IL-6 (431304, BioLegend); Mouse monoclonal antibodies against HA (TA180128, OriGene), Flag (F3165) and β-actin (A2228) (Sigma), IRF3 (sc-33641, Santa Cruz Biotechnology); Rabbit polyclonal antibodies against PGR (8757), SRC (2109), p-SRC^Y419^ (2101) and p-Tyrosine (9411S) (Cell signaling technology), TBK1 (ab40676), p-TBK1^S172^ (ab109272) and p-IRF3^S386^ (ab76493) (Abcam) were purchased from the indicated companies. SeV, EMCV and HSV-1 were previously described.^75,76^ HEK293 cells were obtained from ATCC. The T-47D cells were provided by Dr. Jing Zhang (Wuhan University). The Calu-3 cells were provided by Dr. Ke Lan (Wuhan University).

### Constructs

Flag- and HA-tagged PGR, SRC, IRF3 or its mutants; HA-tagged VISA, TBK1, CRISPR-Cas9 gRNA plasmids for PGR, pSuper-Retro-shRNA plasmids for SRC were constructed by standard molecular biology techniques. The target sequences of genes for gRNA and shRNA construction were listed in Table S1.

### Mice

*Pgr*-knockout mice on the C57BL/6 background were generated utilizing the CRISPR/Cas9 method by GemPharmatech. In *Pgr*^−/−^ mice, the entire fragment containing the first exon of the *Pgr* gene is removed. The strategy for construction of the targeting vector is illustrated in Supplemental Fig. 1a.

Genotyping by PCR was performed using the following pairs of primers.

Primer pair 1: 5′-ATGACACTCAATGTCCAAACTCCC-3′, 5′-TTCCTCAATGCTGGGTCACAAC-3′;

Primer pair 2: 5′-TAGAGCGCCAACGCTTGCTAGA-3′, 5′-TGGTCAGCTCCTGTCCTTACCCTC-3′.

Amplification of the disrupted allele with Primer pair 1 results in a 442-bp fragment, whereas amplification of the WT allele with Primer pair 2 results in a 510-bp fragment.

Mice were maintained in the special pathogen-free facility of Medical Research Institute at Wuhan University. Six- to eight-week-old male mice were randomly allocated for each experimental group and littermates were used as controls. All animal experiments were performed in accordance with the Wuhan University Medical Research Institute Animal Care and Use Committee guidelines.

K18-hACE2 transgenic mice, which express human ACE2 driven by the human epithelial cell cytokeratin-18 (K18) promoter used as an infection model for both SARS-CoV and SARS-CoV-2,^77–79^ were purchased from Gempharmatech and housed in ABSL-3 pathogen-free facilities. All animal experiments were approved by the Animal Care and Use Committee of Wuhan University.

### Transfection and reporter assays

HEK293 cells were transfected by standard calcium phosphate precipitation method. In reporter assays, pRL-TK (Renilla luciferase) reporter plasmid (0.01 μg) was added to each transfection for normalization of transfection efficiency. Luciferase assays were performed using a Dual-Specific Luciferase Assay Kit (Promega). Firefly luciferase activities were normalized on the basis of *Renilla* luciferase activities.

### qPCR

Total RNA was isolated for qPCR analysis to measure mRNA levels of the indicated genes. Data shown are the relative abundance of the indicated mRNA normalized to that of GAPDH. qPCR primers were listed in Table S1.

### Coimmunoprecipitation and immunoblotting analysis

Cells were lysed in NP-40 lysis buffer (20 mM Tris-HCl pH 7.4, 150 mM NaCl, 1 mM EDTA, 1% NP-40,10 μg/ml aprotinin, 10 μg/ml leupeptin, 1 mM phenylmethylsulfonyl fluoride). For each immunoprecipitation, a 0.4 ml aliquot of the lysate was incubated with the indicated antibody or control IgG (0.5 μg, or 0.5 μl for antiserum) and 25 μl of a 1:1 slurry of Protein G Sepharose (GE Healthcare) for 2 h. Sepharose beads were washed three times with 1 ml of lysis buffer containing 0.5 M NaCl. Proteins were separated by 8% SDS–PAGE, followed by immunoblotting analysis with the indicated antibodies.

### Phosphorylation site identification by mass spectrometry

HEK293 cells were transfected with Flag-IRF3 and HA-SRC or Vector. Flag-IRF3 was immunoprecipitated and desalted. The mass spectrometry analysis was performed as previously described by SpecAlly (Wuhan) Life Science and Technology Company.^80^

### Preparation of anti-phospho-IRF3^Y107^

Preparation of anti-Phospho-IRF3^Y107^ was conducted by ABclonal Technology Company. Briefly, a peptide with DPHKI(Y-P)EF-C sequence was synthesized and conjugated to KLH for immunization of the experimental Japanese white-eared rabbits. Rabbit anisera were passed through affinity columns conjugated with the synthesized control peptide (DPHKIYEF-C) for two times. The sera were then purified by affinity columns conjugated with the modified peptide DPHKI(Y-P)EF-C. The final eluted anti-phospho-IRF3^Y107^ was validated by dot blotting analysis with both the synthesized un-modified and modified peptides.

### In vivo IFNAR blockade

For SeV replication experiments, WT mice were intraperitoneally (i.p.) administrated with anti-mouse IFNAR-1 (500 μg/mouse) or PBS, and then injected intravenously with DMSO or P4 (30 μg/kg). Half an hour later, mice were intraperitoneally injected with SeV. Fifteen hours after the first injection of anti-mouse IFNAR-1, the mice were administrated with anti-mouse IFNAR-1 or PBS again (500 μg/mouse). Four hours later, the mice were killed by cervical dislocation for analysis.

For EMCV replication experiments, WT mice were i.p. administrated with anti-mouse IFNAR-1 (500 μg/mouse) or PBS, and then injected intravenously with DMSO or P4 (30 μg/kg). Half an hour later, mice were i.p. injected with EMCV (1 × 10^8^ pfu). The mice were administrated with anti-mouse PBS or anti-mouse IFNAR-1 for two more times, one (500 μg/mouse) or two days (250 μg/mouse) after EMCV infection. The mice were also administrated intravenously with DMSO or P4 (30 μg/kg) for one more time at 2 days after EMCV infection. The mice were killed by cervical dislocation one day later.

### Patients

This study was reviewed and approved by the Medical Ethical Committee of Zhongnan Hospital of Wuhan University (Approval# 2020033-1 and 2020068). The clinical sex-related hormone-testing reports were collected from Wuhan Leishenshan Hospital or Zhongnan Hospital. A convenient sampling strategy was employed, in which all testing reports from consecutive patients meeting inclusion criteria were collected for analysis at the day of their initial visits to the hospitals. We collected sex-related hormone-testing reports from 104 reproductive-aged (median age 40 years, range 31–49) male COVID-19 patients, who were admitted to Wuhan Leishenshan Hospital or Zhongnan Hospital from March 5 to March 31, 2020. The control group were from males who were admitted for evaluation of fertilization ability. A total of 107 age-matched men (median age 40, range 32–49) were randomly selected and the data of their sex hormones were obtained. The diagnosis of COVID-19 and the degree of severity (mild, moderate, severe, critical) were determined according to the New Coronavirus Pneumonia Prevention and Control Program (7th edition) published by the National Health Commission of China.

### Statistics

For mouse experiments, no specific blinding method was used, but mice in each group were selected randomly. The sample size (n) of each experimental group is described in the corresponding figure legend. GraphPad Prism software was used for all statistical analyses. Quantitative data in histograms are shown as means ± s.d. Data were analyzed using a one-way ANOVA, Student’s unpaired *t*-test or Log-rank (Mantel-Cox) test. The number of asterisks represents the degree of significance with respect to P values. Statistical significance was set at *P* < 0.05.

## Supplementary information

Supplementary Materials

## Data Availability

All the data shown in this paper are available from the corresponding authors upon reasonable request.
