# Supplementary material for: Modulation of innate immune response to viruses including SARS-CoV-2 by progesterone
Source: Signal Transduct Target Ther. 2022 Apr 25;7:137. doi: 10.1038/s41392-022-00981-5 (PMC9035769; doi:10.1038/s41392-022-00981-5)
Supplement: Supplementary file 1 — Supplementary Materials [file 41392_2022_981_MOESM1_ESM.pdf]

Supplementary Materials for

Modulation of innate immune response to viruses including SARS-CoV-2 by progesterone

Shan Su, Duo Hua, Jin-Peng Li, Xia-Nan Zhang, Lei Bai, Li-Bo Cao, Yi Guo, Ming Zhang, Jia-Zhen Dong, Xiao-Wei Liang, Ke Lan, Ming-Ming Hu* and Hong-Bing Shu*

*Correspondence to:

Dr. Ming-Ming Hu (mmhu@whu.edu.cn)
Dr. Hong-Bing Shu (shuh@whu.edu.cn)

This PDF file includes:

Figures. S1 to S6
Table S1

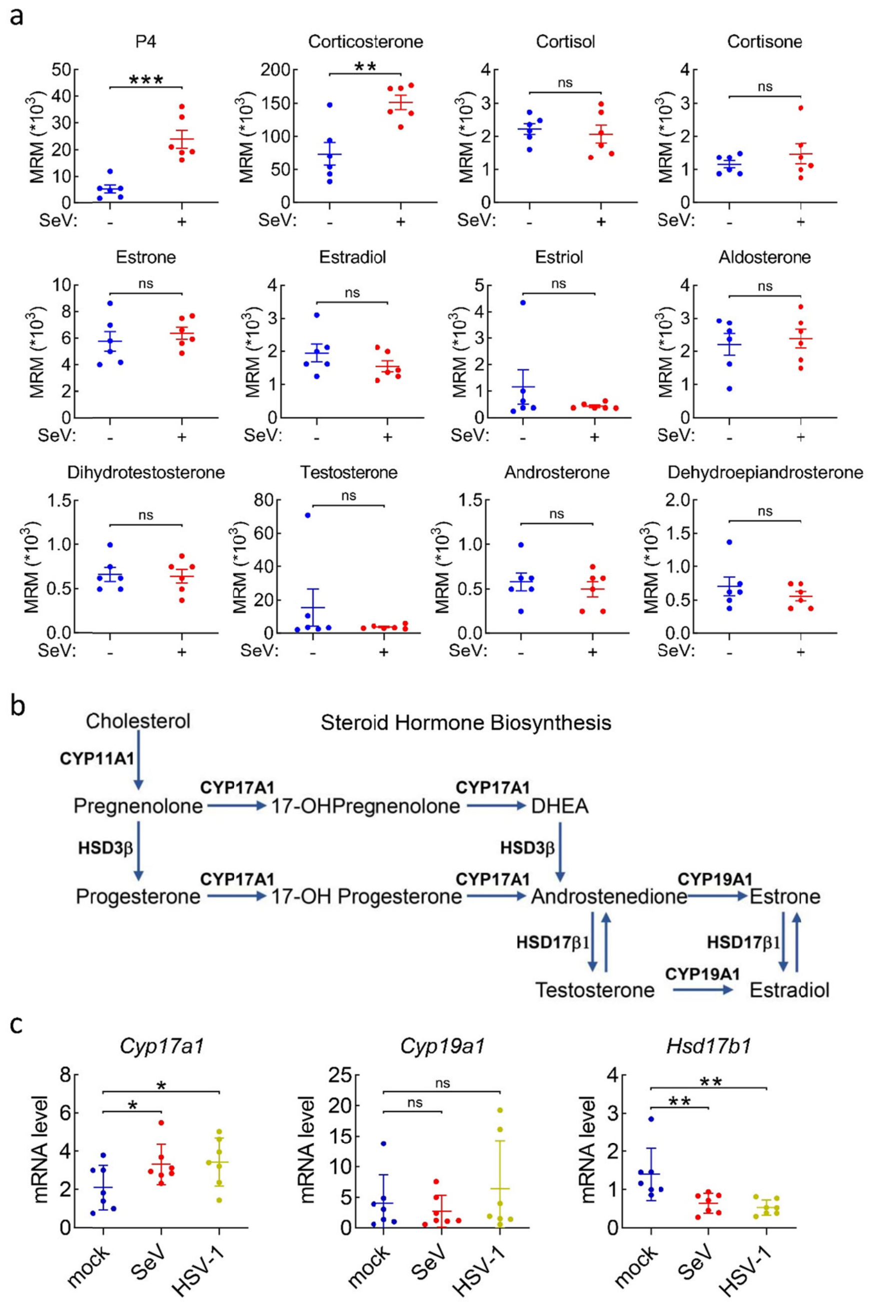

Fig. S1 Viral infection affects metabolic hemostasis of steroid hormones in mice.

a. Targeted metabolomic analysis of steroid hormones in sera of SeV-infected mice.

b. Pathways of steroid hormone biosynthesis.

c. Effects of viral infection on gene transcription of enzymes involved in steroid hormone biosynthesis. C57BL/6 male mice (n=7 in each group) were left un-infected or infected intravenously with SeV for 3 hours or HSV-1 for 6 hours before their adrenal glands were collected for qPCR analysis of mRNA levels of the indicated genes.

*P < 0.05; **P < 0.01; ***P<0.001; ns, not significant.

a One-way ANOVA for IFN-β

SeV	Sum of squares	df	Mean square	F	P value
Between groups	6.724	2	3.362	18.83	P<0.0001
Within groups	2.678	15	0.1785		
Total	9.402	17			

EMCV	Sum of squares	df	Mean square	F	P value
Between groups	23.25	2	11.62	5.048	P=0.0211
Within groups	34.54	15	2.303		
Total	57.79	17			

b

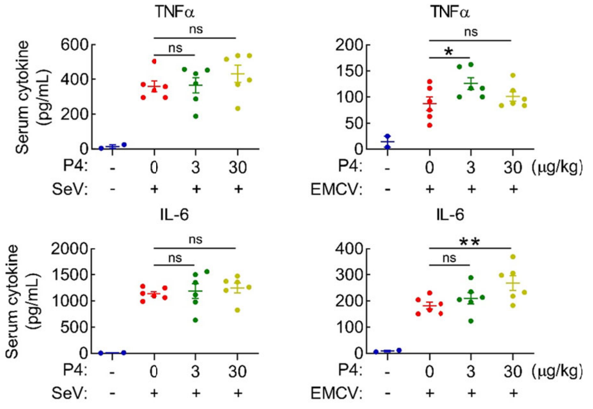

c One-way ANOVA for TNFα

SeV	Sum of squares	df	Mean square	F	P value
Between groups	18999	2	9499	0.8767	P=0.4364
Within groups	162529	15	10835		
Total	181528	17			

EMCV	Sum of squares	df	Mean square	F	P value
Between groups	4660	2	2330	3.003	P=0.08
Within groups	11641	15	776		
Total	16301	17			

d One-way ANOVA for IL-6

SeV	Sum of squares	df	Mean square	F	P value
Between groups	39116	2	19558	0.3111	P=0.7372
Within groups	942908	15	62861		
Total	982024	17			

EMCV	Sum of squares	df	Mean square	F	P value
Between groups	23057	2	11529	4.006	P=0.0404
Within groups	43166	15	2878		
Total	66223	17			

Fig. S2 Progesterone does not affect TNFα and IL-6 production in virus-infected mice.

a. One-way ANOVA analysis for virus-induced IFN-β production in control and P4-treated mice.

b-d. Effects of progesterone on virus-induced inflammatory cytokine production in the sera of mice. Mice (male, n=6 in each group) were injected intravenously with the indicated doses of P4 for half an hour, and then infected with SeV or EMCV (1×10^8 pfu) for 6 hours before measurement of the indicated serum cytokines by ELISA assays. The mock-infected mice (male, n=2) were used as a control. One-way ANOVA analysis for virus-induced TNFα (b) and IL-6 (c) production were performed. *P < 0.05; **P < 0.01; ***P<0.001; ns, not significant.

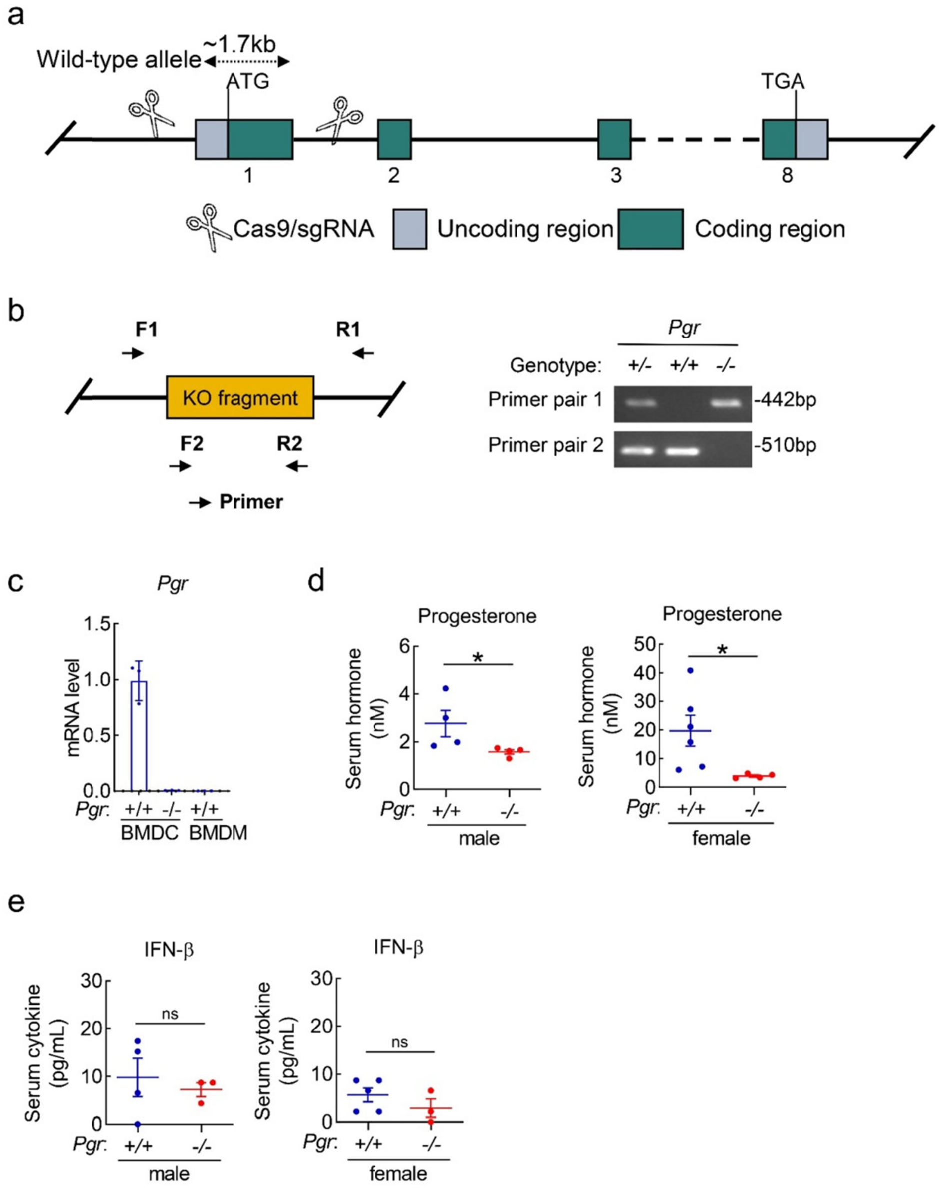

Fig. S3 Generation and characterization of PGR-knockout mice.

a. The strategy for knockout of PGR by the CRISPR-Cas9 method.

b. Genotyping of PGR-knockout mice by PCR.

c. Analysis of the mRNA levels of *Pgr* in BMDCs and BMDMs from the wild-type and *Pgr*-knockout mice. Data shown are mean±SD (n=3).

d-e. Effects of Pgr-deficiency on serum progesterone and IFN-β levels in male and female mice. Serum of *Pgr*$^{+/+}$ (n=4 for male, n=6 for female) and *Pgr*$^{-/-}$ (n=4 for male, n=4 for female), mice were collected from the orbit for analysis of progesterone (d) and IFN-β (e) levels by ELISA. Data from mice with undetectable IFN-β levels are not shown in the histographs. *P < 0.05; **P < 0.01; ***P<0.001; ns, not significant.

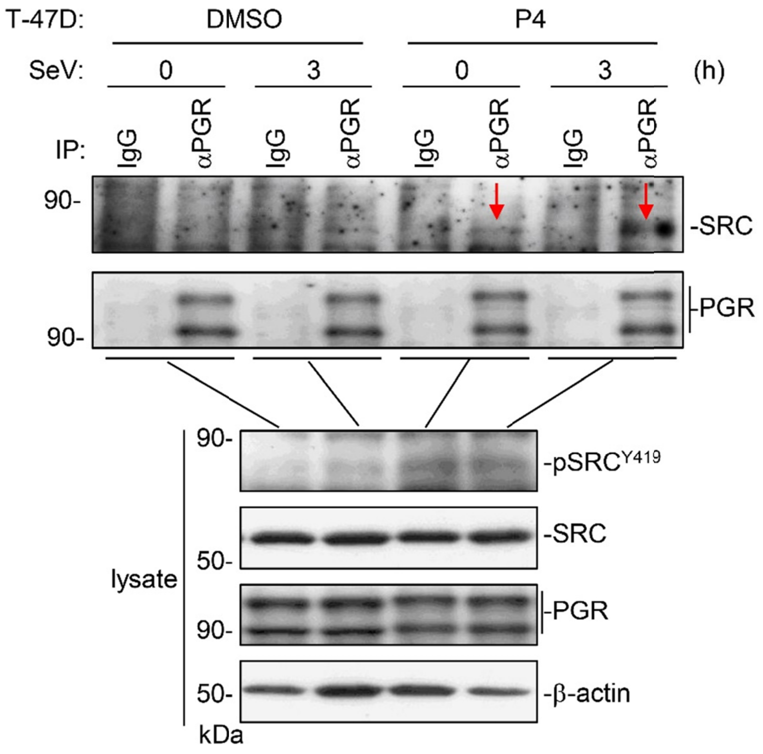

Fig. S4 Endogenous association of PGR with SRC.
T-47D cells were treated with DMSO or P4 (1 μM) for 1 hour, and then left un-infected or infected with SeV for the indicated times before coimmunoprecipitation and immunoblotting analysis with the indicated antibodies.

a

Phosphorylated Residues by Mass Spectrometry

	Site	Sequence and Phospho (STY)	Conservation
IRF3	Y107	IY(1)EFVNSGVGDFSQPDTSPDT	Hu, Mus
	Y292	HCHTY(1)WAVSEELLPNSGHGP	Hu

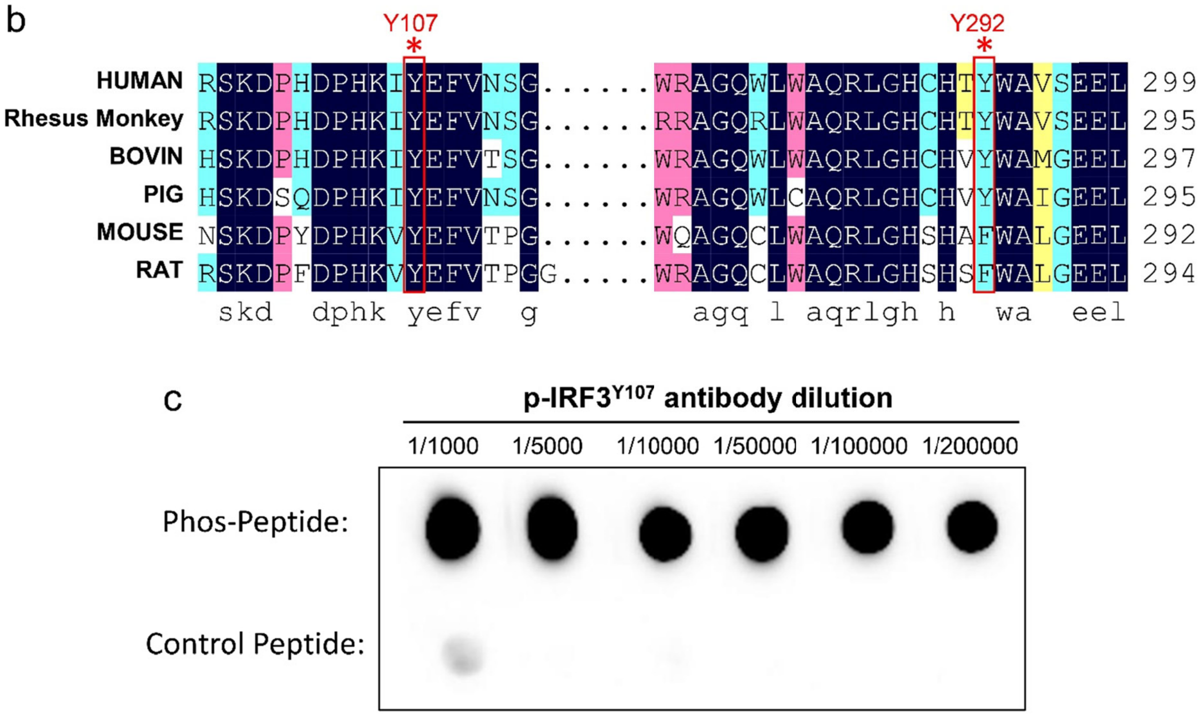

Fig. S5 Preparation of an antibody specific for Y107-phosphorylated IRF3.

a. Identification of phosphorylated residues of human IRF3 following SRC overexpression by mass spectrometry (MS).

b. Alignment of IRF3 partial sequences from different mammalian species.

c. Dot blot detection of the synthetic Y107-phoshorylated and un-phosphorylated IRF3 peptides by the p-IRF3^{Y107} antibody raised against the synthetic phosphorylated IRF3 peptide. The p-IRF3^{Y107} antibody was diluted at the indicated ratios, and then subjected to dot blot analysis.

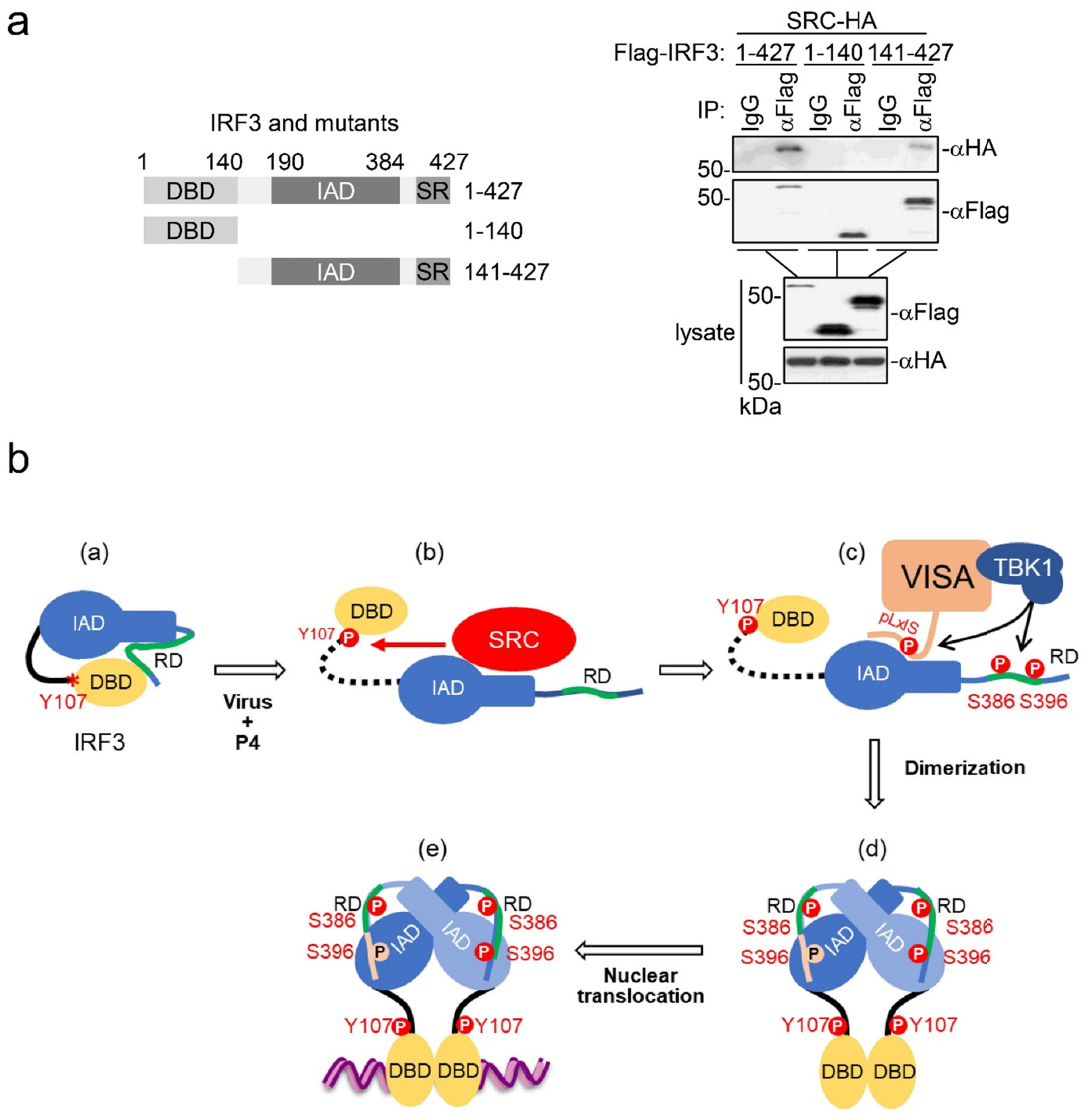

Fig. S6 A Tyrosine phosphorylation of IRF3 Y107 regulates innate antiviral response.

a. Domain mapping of SRC and IRF3. HEK293 cells were transfected with the indicated expression plasmids before co-immunoprecipitation and immunoblotting analysis with the indicated antibodies.

b. A model on how tyrosine phosphorylation of IRF3 Y107 unlocks its intramolecular autoinhibition and primes it for activation.

Table S1. Sequences for gRNA and shRNA and qPCR primers

PGR-gRNA-#1	5'-CCGCGGAGTTCACCCTGTGC-3'
PGR-gRNA-#2	5'-CAATACAGCTTCGAGTCATT-3'
PGR-gRNA-#3	5'-GTCTTAATCAACTAGGCGAG-3'
SRC-shRNA-1#	5'-GCCTCTCAGTGTCTGACTT-3'
SRC-shRNA-2#	5'-GACTTCGACAACGCCAAGG-3'
Human *GAPDH*	5'-GAGTCAACGGATTTGGTCGT-3'
	5'-GACAAGCTTCCCGTTCTCAG-3'
Human *IFNB1*	5'-TTGTTGAGAACCTCCTGGCT-3'
	5'-TGACTATGGTCCAGGCACAG-3'
Human *ISG56*	5'-GCCTTGCTGAAGTGTGGAGGAA-3'
	5'-ATCCAGGCGATAGGCAGAGATC-3'
Human *CXCL10*	5'-GGTGAGAAGAGATGTCTGAATCC-3'
	5'-GTCCATCCTTGGAAGCACTGCA-3'
Human *IRF1*	5'-AGAGCAAGGCCAAGAGGAAGTCAT-3'
	5'-AAGTCCTGCATGTAGCCTGGAACT-3'
Human *CYP11A1*	5'-TGGCATCCTCTACAGACTCCTG-3'
	5'-CTTCAGGTTGCGTGCCATCTCA-3'
Human *HSD3B2*	5'-CGCCTGTATCATTGATGTCTTTGG-3'
	5'-CTGGTGTAGATGAAGACTGGCAC-3'
Human *SGK*	5'-GACCCCGAGTTTACCG AAGAG-3'
	5'-GGAAAGCCTCGGCAGCTT-3'
Human *FKBP54*	5'-GCTCTGGAGAAAATGCAGCGGGA-3'
	5'-TGCATAGGGACTCACACACCTTGA-3'
Murine *Gapdh*	5'-ACGGCCGCATCTTCTTGTGCA-3'
	5'-ACGGCCAAATCCGTTCACACC-3'
Murine *Ifnb1*	5'-TCCTGCTGTGCTTCTCCACCACA-3'
	5'-AAGTCCGCCCTGTAGGTGAGGTT-3'
Murine *Isg56*	5'-TACAGGCTGGAGTGTGCTGAGA-3'
	5'-CTCCACTTTCAGAGCCTTCGCA-3'
Murine *Cxcl10*	5'-ATCATCCCTGCGAGCCTATCCT-3'
	5'-GACCTTTTTTGGCTAAACGCTTTC-3'
Murine *Irf1*	5'-TCCAAGTCCAGCCGAGACACTA-3'
	5'-ACTGCTGTGGTCATCAGGTAGG-3'
Murine *Cyp11a1*	5'-TGCTCAACCTGCCTCCAGACTT-3'
	5'-ACTGGCTGAAGTCTCGCTTCTG-3'
Murine *Hsd3b2*	5'-ATCAGGGTCCTGGACAAGGTCT-3'
	5'-TGGCAAGCTCTCCTCAGGTACT-3'
Murine *Cyp17a1*	5'-AGCTCTGTGCTGAACTGGATCC-3'
	5'-AGACGGTGTTCGACTGAAGCCT-3'
Murine *Cyp19a1*	5'-CGAAGCAGCAATCCTGAAGGAG-3'

	5’-CCAAGTCCACAACAGGCTGGTA-3’
Murine *Hsd17b1*	5’-TTCTGCCAGACATGAAGAGGCG-3’
	5’-CGCAAACTTGCTGGCACAGTAC-3’
SeV genome	5’-GCTCCGGATCGTTACCCATA-3’
	5’-TCATTCCCTGTCTCAGCCTG-3’
EMCV genome	5’-TCTTGGCCGCTTTGTCTAGA-3’
	5’-TGGCTTGGTCTCGACTAGTG-3’
SARS-CoV-2-E	5’-ACAGGTACGTTAATAGTTAATAGCGT-3’
	5’-ATATTGCAGCAGTACGCACACA-3’
SARS-CoV-2-N	5’-TAATCAGACAAGGAACTGATTA-3’
	5’-CGAAGGTGTGACTTCCATG-3’
Taqman probe	
SARS-CoV-2-E	5’-ACACTAGCCATCCTTACTGCGCTTCG-3’
SARS-CoV-2-N	5’-GCAAATTGTGCAATTTGCGG-3’
Human *GAPDH*	5’-CTGCTTAGCACCCCTGGCCA-3’
